# Unveiling new anti-inflammatory mechanisms of nanomaterials

**DOI:** 10.1093/nsr/nwad264

**Published:** 2023-10-10

**Authors:** Zhen Gu

**Affiliations:** College of Pharmaceutical Sciences, National Key Laboratory of Advanced Drug Delivery and Release Systems, Zhejiang University, China; Liangzhu Laboratory, China; Jinhua Institute of Zhejiang University, China; Department of General Surgery, Sir Run Run Shaw Hospital, School of Medicine, Zhejiang University, China; MOE Key Laboratory of Macromolecular Synthesis and Functionalization, Department of Polymer Science and Engineering, Zhejiang University, China

Inflammasome activation is instrumental in clearance of pathogens and damaged cells, playing a pivotal role in maintaining host homeostasis. Nonetheless, it is important to note that inflammasome activation can also be a primary driving factor in autoimmune disease and metabolic dysregulation, encompassing conditions such as Alzheimer's disease, colitis, type 2 diabetes, gout, and atherosclerosis [1]. Macrophages serve as the main cell type responsible for inflammasome activation and the mediation of pro-inflammatory cytokine release. To date, several small molecules have been discovered as effective inhibitors of inflammasome activation in macrophages to alleviate the pathological development of inflammatory diseases [2]; however, these small molecule drugs do not exhibit cell specificity.

Notably, multiple studies have indicated that macrophages readily uptake nanoparticles, leading to the activation of the inflammasome [3,4]. This discernible propensity for macrophage interaction has spurred substantial research endeavors within the field of nanomedicine, thereby indicating the potential for nanoparticles to surpass the efficacy of small molecule inhibitors due to their preferential uptake and processing by macrophages. A novel approach involving cationic lipid-assisted pegylated-poly(lactic-co-glycolic acid) nanoparticles (referred to as CLAN) has demonstrated its effectiveness in delivering Cas9 mRNA (mCas9) and guide RNA (gRNA) into macrophages. This innovative technique has resulted in the precise modification of the *NLRP3* gene, effectively restraining inflammasome activation [5]. This development holds promise in the targeted and highly effective administration of therapeutic agents. Regrettably, numerous prior studies in the field of nanomedicine have documented that the majority of reported inorganic nanomaterials can induce excessive activation of inflammasomes [6,7]. These findings underscore the need for continued research to ensure the safety and effectiveness of nanomaterials while avoiding inflammasome activation in therapeutic applications.

**Figure 1.**
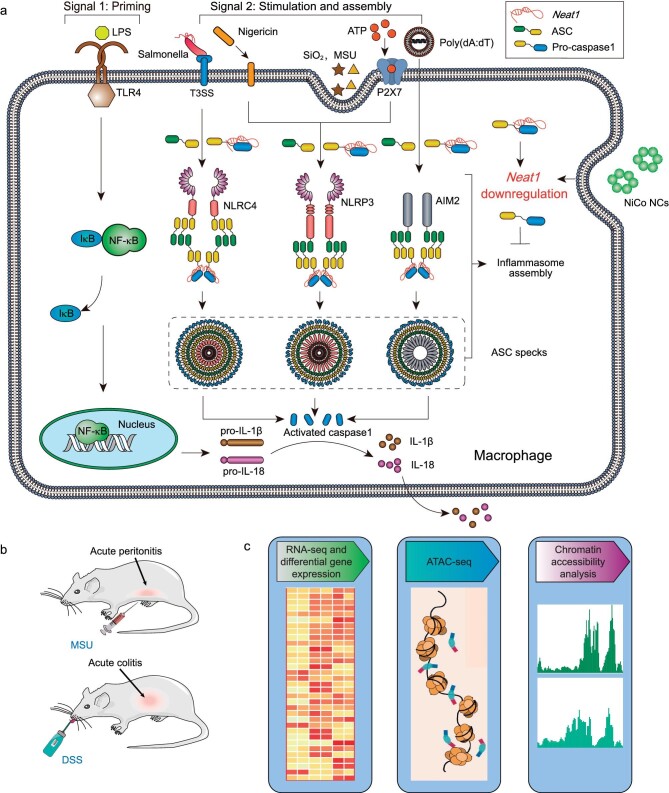
(a) Schematic of inhibition of *NLRP3, NLRP4* and *AIM2* inflammasome activation by NiCo NCs. (b) NiCo NCs suppressed *NLRP3* inflammasome activation in acute peritonitis and colitis mouse models. (c) The exploration of novel anti-inflammatory mechanisms of NiCo NCs by bioinformatics. Reprinted with permission from Ref [8].

A collaborative team from the University of Science and Technology of China, and Hefei University of Technology, has unveiled a new anti-inflammatory mechanism mediated by nanomaterials [8]. They demonstrated that nickel-based and cobalt-based nanomaterials exerted broad-spectrum suppression of *NLRP3, NLRC4*, and *AIM2* inflammasome activation (Fig. 1a). This finding stands in stark contrast to numerous prior research reports that have indicated inorganic nanoparticles typically induce inflammasome activation, highlighting a significant disparity. Moreover, in disease animal models, nickel-cobalt alloy nanocrystals have been confirmed to substantially alleviate symptoms of colitis and acute peritonitis (Fig. 1b).

Unveiling the biological mechanisms underlying the actions of nanomaterials is crucial for their potential medical applications. However, elucidating the biological mechanisms by which these nanocrystals exert broad-spectrum inhibitory effects on inflammasome activation remains challenging through conventional biological experimental approaches. Consequently, the researchers turned to genomic and bioinformatics strategies. Through the utilization of transcriptomic sequencing (RNA-seq), the researchers have demonstrated that this inhibitory effect is realized through downregulating *Neat1*, a long noncoding RNA known to exert a significant role in inflammasome activation [9]. By conducting chromatin accessibility profiling employing ATAC-seq, the researchers further revealed that this inhibitory impact is achieved by reducing the accessibility of the gene body and promoter region of *Neat1*. This alteration signifies the repression of *Neat1* transcription (Fig. 1c).

In summary, this study has revealed novel attributes of inorganic nanoparticles—a broad-spectrum inhibition of inflammasome activation. In addition, this study harnessed the interdisciplinary fusion of nanomedicine and bioinformatics to elucidate the new mechanism by which nickel-cobalt inhibits inflammasome activation through the modulation of expression of a long non-coding RNA. From the perspective of clinical translation, this discovery also offers new insights for designing nanomedicines with enhanced biocompatibility.
